# Variation in Vector Competence for Dengue Viruses Does Not Depend on Mosquito Midgut Binding Affinity

**DOI:** 10.1371/journal.pntd.0001172

**Published:** 2011-05-17

**Authors:** Jonathan Cox, Heidi E. Brown, Rebeca Rico-Hesse

**Affiliations:** 1 Virology and Immunology, Southwest Foundation for Biomedical Research, San Antonio, Texas, United States of America; 2 School of Geography and Development, University of Arizona, Tucson, Arizona, United States of America; Children's Hospital Oakland Research Institute, United States of America

## Abstract

**Background:**

Dengue virus genotypes of Southeast Asian origin have been associated with higher virulence and transmission compared to other genotypes of serotype 2 (DEN-2). We tested the hypothesis that genetic differences in dengue viruses may result in differential binding to the midgut of the primary vector, *Aedes aegypti*, resulting in increased transmission or vectorial capacity.

**Methodology/Principal Finding:**

Two strains of each of the four DEN-2 genotypes (Southeast Asian, American, Indian, and West African) were tested to determine their binding affinity for mosquito midguts from two distinct populations (Tapachula, Chiapas, Mexico and McAllen, Texas, USA). Our previous studies demonstrated that Southeast Asian viruses disseminated up to 65-fold more rapidly in *Ae. aegypti* from Texas and were therefore more likely to be transmitted to humans. Results shown here demonstrate that viruses from all four genotypes bind to midguts at the same rate, in a titer-dependent manner. In addition, we show population differences when comparing binding affinity for DEN-2 between the Tapachula and McAllen mosquito colonies.

**Conclusions:**

If midgut binding potential is the same for all DEN-2 viruses, then viral replication differences in these tissues and throughout the mosquito can thus probably explain the significant differences in dissemination and vector competence. These conclusions differ from the established paradigms to explain mosquito barriers to infection, dissemination, and transmission.

## Author Summary

Several factors, such as mosquito and virus genetics and environmental variables, determine the ability of mosquitoes to transmit dengue viruses. In this report, we describe new and important information that in some ways contradicts what is in the literature. Midgut infection barriers have been described as important determinants of virus transmission in mosquitoes but we found that virus binding to these midgut cells does not vary. When we compared binding of 8 different, low passage dengue viruses to mosquito midguts that were dissected out of *Aedes aegypti* mosquitoes (the main vectors of dengue) from Mexico and Texas, we found that there were no differences. Previously, we (and others) had shown that these same viruses differed significantly in replication and dissemination throughout the rest of the mosquito body, including the salivary glands, and therefore they differed greatly in their potential to be transmitted to humans. Thus, the data presented here are important considerations for future studies of vector competence and in determining strategies for control of dengue viruses in the vector.

## Introduction

Dengue viruses, which cause millions of cases of dengue fever (DF) and dengue hemorrhagic fever (DHF) each year in over 100 countries, are transmitted by two species of mosquitoes, *Aedes aegypti* and *Aedes albopictus*. These vectors directly determine the rates of transmission of dengue viruses to humans, and they therefore determine the global spread and occurrence of disease. Thus, understanding the factors that make mosquitoes susceptible to infection by dengue viruses could help us design specific methods of control, whether these methods affect the mosquitoes directly (e.g. genetic manipulation) or indirectly (e.g. population dynamics). The factors that influence the mosquito's capability for virus transmission, or vector competence, are numerous and have been reviewed in detail [1], [2]. Vector competence is defined as the intrinsic permissiveness of a vector to infection, replication, and transmission of a virus. Specific factors, including mosquito and viral genetics and the environment, that govern *Ae. aegypti* transmission of dengue viruses (members of the *Flavivirus* family), were reviewed recently [3]; however, it is important to point out that other flaviviruses, such as yellow fever and West Nile differ in their interactions with this mosquito. Gubler and Rosen were the first to study dengue vector competence, in both *Aedes* species, by comparing growth of representatives of the four different serotypes [4], [5]; they were the first to describe a “gut barrier” for dengue virus dissemination in these mosquitoes. That is, dengue virus serotypes differed in their ability to escape two anatomical barriers to infection, the midgut infection barrier and the midgut escape barrier. The midgut barrier involves the ability of a virus to infect and replicate in midgut cells, with a variation in the ability to bind cell surface receptors or these cells not replicating virus (non-permissiveness). The midgut escape barrier hinders the ability of virus to exit and disseminate to other tissues despite viral replication in the midgut, even to high titers. A barrier to virus transmission in the salivary glands was described in 1976 [6] and involves infection of those cells with no, or undetectable, virus being released in the saliva when mosquitoes bite.

In contrast with most studies of vector competence, we have focused on comparing infection, replication, and transmission rates of dengue viruses in field-collected mosquitoes, with generations from eggs not higher than F4 (i.e., not lab-adapted mosquito colonies), and by comparing many different, low-passage (i.e., isolated from human patients or sylvatic mosquitoes, not lab-adapted) virus strains of the same serotype (DEN-2). In addition, most of the viruses we used in our studies have been fully characterized, by determining their nucleotide sequences (mostly entire genome or envelope gene only), by measuring their growth in primary human cells (monocyte and dendritic cells, the natural targets of infection in human skin) [7], and by comparing their replication and disease causation (virulence) in a mouse model of dengue fever [8]. Thus, we have compared *Ae. aegypti* vector competence for a very broad spectrum of genetic variants belonging to one serotype of dengue, and with defined differences in phenotype (growth, virulence, and epidemiologic) and genotype (phylogenetic grouping).

In the experiments described here, we compared mosquito *ex-vivo* midgut binding of viruses belonging to the four genotypes of DEN-2 [9] from females of two mosquito colonies from different locations (Tapachula, Chiapas, Mexico and McAllen, Texas, USA). Our results, in conjunction with those we reported previously on vast differences in virus infection and dissemination rates in these mosquitoes [10], lead us to conclude that the midgut infection barrier for dengue viruses in *Aedes aegypti* may not be due to viral binding to these cells but rather to differences in viral genome replication ability. That is, differences in mosquito transmission abilities are probably determined by viral genetics and these determinants must be included in any attempts to control dengue virus transmission.

## Materials and Methods

### Virus Strains

Eight DEN-2 virus strains, representing the Southeast Asian, American, Indian, and West African genotypes, were used in this study (Table 1). All viruses were low passage (eight or less) isolates from either patients or mosquitoes in order to reduce mutations associated with culture adaptation. Virus stocks were prepared as follows: the *Aedes albopictus* larval-derived cell line, C6/36, was grown to 90% confluency in 175-cm^2^ flasks using growth media (minimal essential medium, 10% fetal bovine serum [FBS, Atlanta Biologicals], 2 mM L-glutamine, 1× non-essential amino acids, 100 u/mL of penicillin, and 100 µg/mL of streptomycin), then inoculated with dengue virus at a multiplicity of infection of approximately 10 genome equivalents/cell in 2 mL of maintenance media (minimal essential medium, 2% FBS, 2 mM L-glutamine, 1× non-essential amino acids, 100 u/mL of penicillin, and 100 µg/mL of streptomycin) and incubated for 1 hour at 28°C in an atmosphere of 5% CO_2_. Flasks were then supplemented with 30 mL of maintenance media and maintained at 28°C in an atmosphere of 5% CO_2_. Infection was monitored daily after 7 days by an indirect fluorescent antibody test (IFAT) and cell supernatants were harvested when more than 90% of the cells expressed dengue viral antigen, no more than 9 days. Individual virus aliquots were stabilized by the addition of 2% Prionex Reagent (Calbiochem) and stored at −70°C.

**Table 1:** Characteristics of the dengue viruses used in this study.

Serotype/Strain	Genotype	Country and year of isolation	Virus titer^a^	Passage history^b^
K0049	SEA	Thailand, 1995	1.46×10^10^	C3
429557	SEA	Mexico, 2005	1.62×10^9^	M1/C2
328298	AM	Mexico, 1995	1.95×10^9^	C3
IQT2913	AM	Peru, 1996	4.82×10^9^	C4
1349	Indian	Burkina Faso, 1982	3.43×10^9^	M1/C6
ArA6894	Indian	Burkina Faso, 1986	1.09×10^10^	S4/C2
DakArA1247	W. African	Ivory Coast, 1980	1.55×10^8^	S5/C3
PM33974	W. African	Republic of Guinea, 1981	1.76×10^9^	M1/C3

a genome equivalents/mL.

b C, C6/36 mosquito cell line; M, mosquito; S, suckling mouse.

### Tissue Culture Infectious Dose 50 (TCID50)

Sterilized glass coverslips were inserted in each well of 6-well plates and seeded with 2×10^6^ C6/36 cells with 2 mL of growth media. After two days, media was removed and replaced with serial dilutions (10^2^–10^6^ genome equivalents) of virus in a total volume of 500 µL of maintenance media and incubated for 1 hour at 28°C in an atmosphere of 5% CO_2_. An additional 2 mL of maintenance media was added and plates were incubated for 7 days at 28°C in 5% CO_2_. An IFAT test was then performed to calculate the strain-specific TCID50.

### Indirect fluorescent antibody test (IFAT)

Immunofluorescence was used to test for infection of C6/36 cells. For viral stock preparation, C6/36 cells were prepared for IFAT by spotting 10 µL of cell suspension onto a multi-well slide and incubated in a humid chamber for 20 minutes at 37°C. Cells were fixed by immersion in ice-cold acetone for 10 minutes. Slides were air-dried and each well was incubated with 20 µL mouse ascitic fluid containing anti-DEN-2 monoclonal antibodies (MAb 3H5, Centers for Disease Control and Prevention) diluted 1∶200 in phosphate-buffered saline (PBS) for 30 minutes at 37°C. Slides were washed twice in PBS followed by incubation of each well using fluorescein isothiocyanate-labeled, goat anti-mouse IgG (Sigma) diluted 1∶200 in PBS for 30 minutes at 37°C. Slides were washed twice in PBS and immediately examined at 200× using a Nikon (Melville) Eclipse E400 microscope with an epi-fluorescence attachment. To determine viral titer by TCID50 the following modifications to the above protocol were made: glass coverslips were removed from six-well slides, immediately fixed in acetone and air dried. Coverslips were attached to glass slides using super glue (Gorilla) and rubber cement (Elmer's) was used to create wells on each coverslip.

### Mosquito Colonies

*Aedes aegypti* mosquito eggs were collected from Tapachula, Chiapas, Mexico and McAllen, Texas, USA during the spring and summer of 2010 to establish colonies. The F2–F3 (Tapachula) and F4 (McAllen) generations were used in this study. Mosquitoes were maintained in an insectary at 26–28°C, a relative humidity of 70–80%, and a 12∶12 hour light-dark cycle. Larvae were hatched and reared in pans of water at a density of 100–300 larvae per liter and fed a mixture of ground rabbit chow (Purina)∶liver powder (Bio-Serv)∶yeast (Bio-Serv) (4∶2∶1) *ad libitum*. Pupae were transferred to screened cages and emergent adults were maintained on an *ad libitum* diet of 10% sucrose (Sigma) in water. Successive Tapachula generations were generated by providing female mosquitoes a 37°C defibrinated rabbit-blood (Colorado Serum Company) meal using a water-jacketed membrane (hog intestine) feeder. Eggs were collected, kept moist for at least 24 hours, and air-dried prior to storage.

### Midgut Isolation and ex-vivo Infection

In contrast to colony mosquitoes, mosquitoes used in experiments were not given blood meals but were maintained on 10% sucrose solution *ad libitum*. Midguts were dissected from 24 hour-starved adult (1–2 week old) female *Ae. aegypti* in cold PBS+5% FBS [11]. Each replicate consisted of two intact midguts, with only the ends cut as a result of dissection with forceps. Midgut pairs were then washed once prior to incubation with virus and centrifuged for 3 minutes to aid in the supernatant removal. Pairs of midguts were incubated with 10^6^–10^8^ genome equivalents (approx. 3,000–300,000 PFU total) of virus in PBS+5% FBS in a total volume of 100 µL at 4°C for one hour. These virus quantities range from what a mosquito might ingest when biting a single viremic human to well above the estimated range (see below). To remove unbound virus from midguts, the supernatant was removed after centrifuging for 3 minutes; 1 mL of fresh 4°C PBS+5% FBS was then added and transferred along with the midguts to a clean 1.5 mL microcentrifuge tube. A total of eight washes were necessary to remove virus not associated with the midguts, before the final RNA extraction.

### Quantitative Reverse Transcription Polymerase Chain Reaction (qRT-PCR)

Total RNA was extracted from 50 µL of viral stocks and midgut tissue using Trizol reagent (Invitrogen), following the manufacturer's instructions. Pelleted RNA was resuspended in a final volume of 50 µL of DEPC-H_2_O. Viral RNA copies in viral stocks and midgut tissue were estimated using a previously reported protocol [12] with some modification. RNA template (10 µL) was amplified in duplicate using an RNA Ultrasense one-step quantitative RT-PCR system (Invitrogen) in a final reaction volume of 25 µL containing 1.25 µL of enzyme mix, 5 µL of 5× Ultrasense reaction mix buffer, 0.5 µL of 5′-carboxy-X-rhodamine reference dye, 5 µM of d2C16A primer (5′-GCTGAAACGCGAGAGAAACC-3′) [forward], 5 µM of d2C46B primer (5′-CAGTTTTAITGGTCCTCGTCCCT-3′) [reverse] and 5 µM of VICd2C38B probe (FAM-5′-AGCATTCCAAGTGAGAATCTCTTTGTCAGCTGT-3′-TAMRA). Amplification was performed on a 7500 real-time PCR system (Applied Biosystems) as follows: one cycle at 48°C for 30 minutes, one cycle at 95°C for 15 minutes and 40 cycles at 95°C for 15 seconds and 60°C for 1 minute. To estimate RNA copy number, a standard curve was generated using in vitro-transcribed RNA standards, as described below.

To directly compare dengue viral copy number in *ex-vivo* infected midguts, the housekeeping gene *Rp17S* was used to quantify midgut tissue following a previously reported protocol [10]. RNA template (10 µL) was amplified in duplicate following the RNA Ultrasense one-step quantitative RT-PCR system described above with the following modifications: The Rp17S specific forward primer (5′-ACATCTGATGAAGCGCCTGC-3′), reverse primer (5′-ACACTTCCGGCACGTAGTTGT-3′), and probe (TET-5′-CACTCCCAGGTCCGTGGTATCTCCATC-3′-TAMRA) replaced dengue specific primers and probe. Viral and *Rp17S* RNA standards were produced as follows: a 94 base pair fragment of the DEN-2 (strain K0049) and a 101 base pair fragment of total mosquito (*Ae. aegypti*) RNA, were amplified by RT-PCR and separately cloned into the pCR2.1 plasmid using TOPO cloning kit (Invitrogen). The recombinant plasmids were linearized with HindIII, and RNA transcripts were generated with a T7 Megascript kit (Ambion) following the manufacturer's instructions. Concentrations of transcribed RNAs were determined with a Ribogreen RNA quantitation kit (Molecular Probes), and 10-fold serial dilutions were prepared and used to construct a standard curve.

### Statistical analyses

For comparison between virus genotypes and mosquito populations we used STATA v. 10 to perform a two-way factorial analysis of covariance (ANCOVA) with interaction. There were 35 mid-gut sample pairs for the American genotype, 36 Indian, 35 Southeast Asian, and 29 West African. There were 69 mid-gut sample pairs for the Tapachula and 66 for the McAllen populations. The dependent variable, number of virus genomes that bound to the midguts, was log_10_ transformed. The independent variables included the log_10_ transformed standardized viral genome equivalents added as the covariate and the four genotypes and two mosquito populations, both categorical. TCID50 s were calculated using the ID50 program, version 5.0 (John L. Spouge, National Center for Biotechnology Information).

## Results

Using the two-way factorial ANCOVA we found no interaction between the virus genotype and the mosquito population when controlling for the standardized viral genome equivalents added (F(3,119) = 1.26, p = 0.291). This indicates that there is no measureable difference in midgut binding for any specific DEN-2 genotype and mosquito population pairings we tested.

### Dengue virus bound to midguts, compared between DEN-2 genotypes

**Figure 1:**
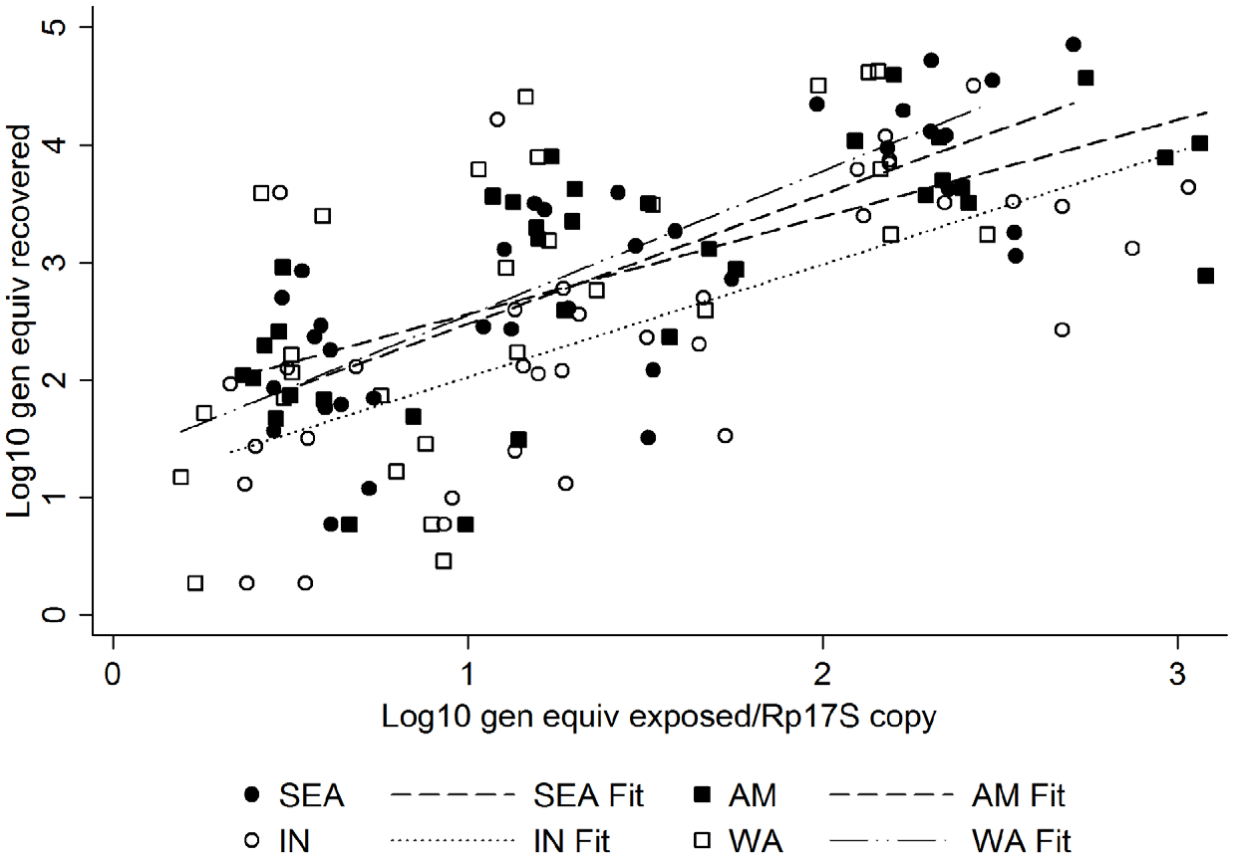
Comparison of DEN-2 virus genotypes in *ex-vivo* midgut infections.

We found no statistically significant difference among the four DEN-2 genotypes in midgut binding affinity (F(3,127) = 0.79, p = 0.501). This indicates that the number of virus genomes that bound to the midgut is not dependent on the viral genotype. The lack of significant differences among genotypes suggests there are no genotype-specific midgut receptors. Moreover, with the range of virus tested, within and exceeding what can be expected when mosquitoes bite a single viremic human (approx. range: 0.1–1,000 PFU per µl) [13]–[17], the virus does not saturate available binding sites. Instead there is a linear relationship between the number of viral genomes that bind to the midguts and the number of viral genomes added to midguts (Figure 1).

### Differences in midgut binding between mosquito populations

**Figure 2:**
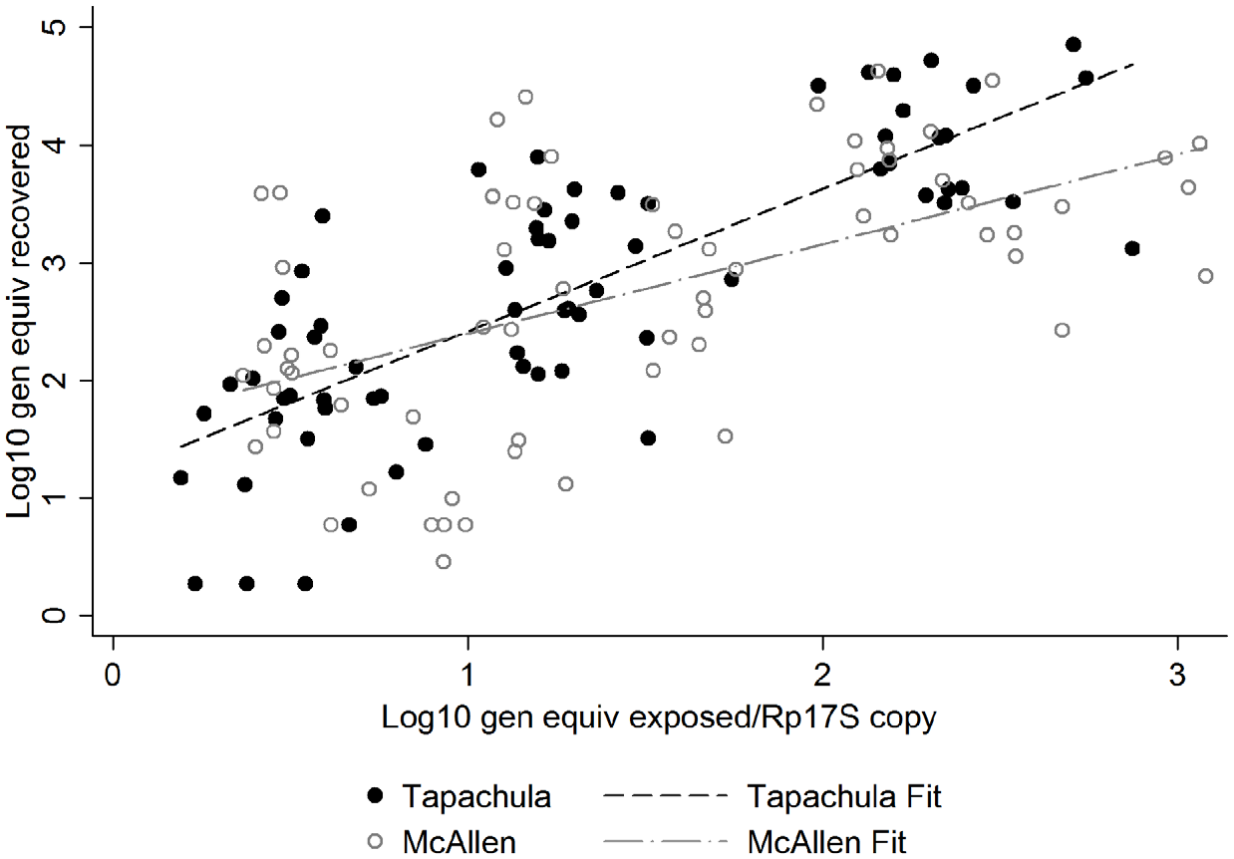
Comparison of Tapachula and McAllen mosquito ex-vivo midgut infections.

When controlling for the standardized viral genome equivalents added, we found a statistically significant difference between the two mosquito populations with respect to the DEN-2 genotypes we tested (F(1,131) = 6.03, p = 0.015). When adjusting for the standardized viral genome equivalents added, the Tapachula mosquitoes bound more virus than mosquitoes from McAllen (Figure 2). This suggests that mosquitoes from the Tapachula site may be somewhat more susceptible to DEN-2 virus infection than mosquitoes from McAllen.

### Differences in Tissue Culture Infectious Dose 50%

Though the midguts were exposed to similar viral dilutions, the relationship between genomic equivalents (RNA) and infectious particles may vary among the genotypes. To determine if this was the case, TCID50 s were calculated for strains belonging to the four genotypes. Two genotypes, Southeast Asian (K0049 TCID50 = 99 genomic equivalents) and American (IQT2913 TCID50 = 150 genomic equivalents), were found to generate or require a log or fewer genomic equivalents than the West African (PM33974 TCID50 = 1110 genomic equivalents) and Indian (ArA6894 TCID50 = 2240 genomic equivalents) genotypes, for infection of C6/36 cells. Differing TCID50 s between genotypes indicates a lack of consistency in the correlation between RNA transcripts measured by RT-PCR and infectious particles, a phenomenon we described previously [18], as there is also a difference in plaque forming ability between DEN-2 strains and genotypes. However, we continue to use RNA equivalents as the best measure of viral quantities for standardization of inputs, and the testing of several dilutions per strain allows us to see this effect, independent of the actual number of RNA strands used for infection.

### Passage histories of viruses did not influence binding

As can be seen in Table 1, we used a range of low passage number dengue virus isolates for these studies. The West African genotype viruses had the highest passage (4 and 8), including replication in suckling mouse brain, whole mosquitoes and cell lines; the Indian genotype viruses were next highest (6 and 7), including similar passage histories. The other two genotypes had the lowest number of passages and were limited to mosquitoes and cell lines. Because we used a range of virus inputs to infect dissected midguts, the trends of binding affinities shown in Fig. 1 reflected no differences between virus genotypes. Thus, there were no statistically significant differences in midgut binding that could be associated with virus passage levels.

## Discussion

In 2006 we reported significant differences in replication and dissemination of DEN-2 viruses of the American and Southeast Asian genotypes in *Ae. aegypti* from Texas [10]. The Southeast Asian genotype viruses had been shown to be associated with epidemics of DHF, while the American genotype viruses had not (typically only DF); the Southeast Asian viruses produced significantly more virus in *ex vivo* infected human dendritic cells, thus probably producing much higher viremias in patients; and these viruses grew and produced significantly higher disease signs in humanized mice, our animal model of DF [8]. Therefore, these characteristics seemed to explain the ecological or epidemiological displacement of the American genotype by the Southeast Asian genotype viruses in this continent and the displacement of the West African and Indian genotypes in those continents (reviewed in [19]). However, the mechanism underlying these differences remained under debate.

Our data reinforce the assertion that the observed differences between genotypes are the result of replication differences and are not due to a variation in midgut tissue affinity. Southeast Asian genotype viruses (3 strains) outcompete American (3 strains) genotype viruses in human dendritic cells and *Ae. aegypti* mosquitoes [20]. Viral replication begins within hours of infection [20], [21] and in this study midguts were incubated with virus for 1 hour. Using orally infected mosquitoes from McAllen and Iquitos, Peru, Armstrong and Rico-Hesse [18] demonstrated that Southeast Asian DEN-2 viruses infected and disseminated much more efficiently than American viruses, when monitored over a two week time course. Similarly, Anderson and Rico-Hesse found the proportion of midguts from orally infected McAllen mosquitoes positive for DEN-2 antigen by IFAT over a two week time course were consistently higher for Southeast Asian viruses compared to American viruses, as were the titers based on quantitative RT-PCR [10]. Southeast Asian strains showed increased disseminated infection and susceptibility in mosquitoes from McAllen and Tehuantepec, Mexico, when compared to American genotype viruses [22]. A possible mechanistic explanation for these differences was provided recently using West Nile virus, a related *Flavivirus*, where virus diversification is driven by the mosquito RNA interference (RNAi) pathway [23]. The RNAi pathway is not activated until virus replicates within cells, a point which is beyond virus binding to cells. The lack of significant differences in DEN-2 genotype binding, as we show here, suggests that little selective pressure is placed on virus infection, in contrast to replication.

Our focus on the first step in infection, virus binding to tissue, is unique and likely helps explain our findings which indicate no infection differences between genotypes. Instead, most researchers have looked at replication or dissemination as endpoint for infection [4], [7], [24], [25], [26]. Bosio et al [25] suggested a midgut infection barrier to explain dengue infection differences between mosquito populations. We find no evidence for an infection barrier based on the results described here. This is not the first time we have found evidence to implicate viral replication over binding affinity in defining infection rates. Though not specifically designed to assess whether virus binding was altered, our previous work showed that recombinant Southeast Asian genotypes lost their increased replication capacity in human cells when the Southeast Asian elements on the envelope glycoprotein (E390) and 5′- and 3′- untranslated regions were replaced by American genotype structures [7].

We detected a significant difference when comparing the DEN-2 infection rates between the two mosquito populations. Finding differences in mosquito population responses to DEN-2 is consistent with those of Gubler et al. [4] and Black et al., as summarized in a 2002 review article [3]. The latter found that mosquitoes from the Pacific coast of Mexico (57%) were more competent as vectors for one strain of DEN-2 (Jamaica 1409) than those from northeastern Mexico and Texas (53%). Likewise, Lambrechts et al. [24] highlight a significant interaction between virus genotype and mosquito population probably explained by co-adaptation. Though we did not see a virus genotype-specific interaction in our investigation of virus binding, we did measure mosquito susceptibility differences. Alternatively, although we did not measure size, the Tapachula mosquitoes appeared smaller than the McAllen mosquitoes in our colonies. Smaller sized *Ae. aegypti* females are more likely to become infected and disseminate virus than larger individuals (based on wing length) [27]. It may be that size differences account for differences in dose response. However, to truly test for mosquito population differences in midgut binding we would need to compare multiple DEN-2 strains binding across more than two mosquito populations.

Our results are consistent with those of one midgut infection study done with genetically-selected *Ae. aegypti* strains, with varying susceptibility to dengue viruses [28]. Two low passage DEN-2 strains (PR-159, an American genotype, and SLK-1592, an Indian genotype) were used to infect two mosquito strains (D2S3 and D2MEB) in order to compare midgut replication and virus dissemination rates. Both mosquito strains were described as possessing high midgut infection rates but D2MEB has a lower dissemination rate compared to D2S3 when challenged with a high passage DEN-2 strain (Jamaica, 1409). Though the authors highlighted their findings using high passage DEN-2 viruses, their low passage viruses showed no differences between the two mosquito populations. That is, when utilizing a more natural experimental protocol, i.e. low passage virus, their findings were similar to ours of no midgut barrier. Unfortunately, all other studies of genetically-controlled determinants of vector competence done by this group of researchers have used only a high passage strain of DEN-2 (Jamaica, 1409) [25], [29], [30], and their results are therefore not comparable to ours. Thus, although we are using dissected midguts, with attempts to maintain the basal/luminal architecture, our results are consistent with other observations on virus replication and dissemination in the entire mosquito, including our own. In summary, we demonstrate that mosquito dissemination and transmission differences observed between DEN-2 genotypes are the result of viral replication and are not due to differences in viral binding to midgut cells.
